# Three-dimensional meniscus allograft sizing—a study of 280 healthy menisci

**DOI:** 10.1186/s13018-020-01591-z

**Published:** 2020-02-24

**Authors:** Silvan Beeler, Lukas Jud, Marco von Atzigen, Reto Sutter, Philipp Fürnstahl, Sandro F. Fucentese, Lazaros Vlachopoulos

**Affiliations:** Department of Orthopaedics, University of Zurich, Balgrist University Hospital, Forchstrasse 340, 8008 Zurich, Switzerland

## Abstract

**Background:**

Inaccurate meniscus allograft size is still an important problem of the currently used sizing methods. The purpose of this study was to evaluate a new three-dimensional (3D) meniscus-sizing method to increase the accuracy of the selected allografts.

**Methods:**

3D triangular surface models were generated from 280 menisci based on 50 bilateral and 40 unilateral knee joint magnetic resonance imaging (MRI) scans. These models served as an imaginary meniscus allograft tissue bank. Meniscus sizing and allograft selection was simulated for all 50 bilateral knee joints by (1) the closest mean surface distance (MeSD) (3D-MRI sizing with contralateral meniscus), (2) the smallest meniscal width/length difference in MRI (2D-MRI sizing with contralateral meniscus), and (3) conventional radiography as proposed by Pollard (2D-radiograph (RX) sizing with ipsilateral tibia plateau). 3D shape and meniscal width, length, and height were compared between the original meniscus and the selected meniscus using the three sizing methods.

**Results:**

Allograft selection by MeSD (3D MRI) was superior for all measurement parameters. In particular, the 3D shape was significantly improved (*p* < 0.001), while the mean differences in meniscal width, length, and height were only slightly better than the allograft selected by the other methods. Outliers were reduced by up to 55% (vs. 2D MRI) and 83% (vs. 2D RX) for the medial meniscus and 39% (vs. 2D MRI) and 56% (vs. 2D RX) for the lateral meniscus.

**Conclusion:**

3D-MRI sizing by MeSD using the contralateral meniscus as a reconstruction template can significantly improve meniscus allograft selection. Sizing using conventional radiography should probably not be recommended.

**Trial registration:**

Kantonale Ethikkommission Zürich had given the approval for the study (BASEC-No. 2018-00856).

## Background

The meniscus plays an important role in the kinematics of the knee joint, reduces contact pressure [1], and improves joint stability [2, 3]. These functions disappear after subtotal or total meniscectomy with resultant early osteoarthritis [4].

Meniscus allograft transplantation seems to be a valuable option for pain reduction and improvement of function in patients with (chronic) postmeniscectomy syndrome [5]. The first meniscus allograft transplantation was performed in 1989 [6] and has been widely used with encouraging results. Several studies demonstrated good or excellent short- to medium-term results [6–12]. However, while midterm survivorship is reported to be 85–90%, long-term survivorship decreases to 50–70% [13].

Reconstruction of a geometrically similar meniscus seems to be crucial for physiological joint pressure distribution [14–21] and good clinical results [22–25]. Undersized grafts could lead to excessive loads due to poor congruity with the femoral condyle, while oversized grafts lose their function by extrusion from the compartment [14]. A mismatch of a few millimeters is supposed to result in poorer biomechanical outcomes [17, 19, 26–29] and increased degenerative changes [18, 30]. Therefore, sizing should be as close to the native meniscus as possible.

Different methods have been described to determine the size of a meniscus [4, 31–35]. Today, meniscus sizing is most commonly performed by conventional radiography according to the Pollard method [31] or by magnetic resonance imaging (MRI) scans [32, 33], and the latter appears to be superior [33, 36]. Unfortunately, inaccuracy of sizing is still a relevant problem in allograft surgery by these methods and has to be improved [17, 25, 31, 36–39].

3D meniscus sizing was recently proposed as a solution to increase the accuracy and precision of meniscus allograft selection [40]. The contralateral meniscus can be used as a very precise reconstruction template. However, advantages of 3D-MRI sizing with the contralateral meniscus as a reconstruction template compared with existing sizing methods have not been shown to date. We hypothesized that 3D-MRI sizing with the contralateral side is clearly superior compared with the currently used sizing methods.

## Methods

The following description of the approaches used for the validation of the different sizing methods is divided into three parts. Part 1 focuses on describing the basic material, imaging, and measurement methods. Part 2 is dedicated to explaining the three different sizing methods in detail. Finally, part 3 contains the sizing validation methods.

### Part 1: Material/imaging/measurements

#### Material

Fifty patients with bilateral and 40 patients with unilateral complete imaging (34 men, 56 women; mean age 26.7 years (range 15–50)) were retrospectively included in this study. All patients had a patellofemoral disorder. The inclusion criteria were patients with available MRI scan and conventional radiography, mature skeletal age, healthy contralateral meniscus, and no tibio-femoral osteoarthritis (Kellgren and Lawrence grade 0) [41]. The presence of a completely closed growth plate at the distal femur and the proximal tibia in the MRI images was used to determine mature skeletal age. Meniscus integrity was assessed by a fellowship-trained musculoskeletal radiologist, and the presence of meniscus tears, degeneration, or extrusion led to exclusion.

#### Imaging

All radiographs (RX) were performed in a standard fashion, with a plain anteroposterior (AP) and lateral (LAT) view with the ampoule placed 1 m distant from the knee using calibrators for the correction of magnification (Optimus 50 X-ray Generator; Philips, USA).

All MRI scans were performed in our institution on a 3.0-T scanner (Skyra-fit, Siemens Healthineers, Erlangen, Germany) with a send/receive knee coil, and the patient was examined in the supine position. All MRI examinations consisted of sagittal, coronal, and axial sequences as part of the standard MRI protocol [40]: (1) coronal short tau inversion recovery (STIR) sequence (repetition time 4200 ms; echo time 34 ms; inversion time 210 ms; slice thickness 3 mm; number of slices 23; bandwidth 245 Hz/pixel; flip angle 150°; matrix 384 × 384; field of view 16 cm); (2) sagittal intermediate-weighted sequence with the Dixon technique (repetition time 4200 ms; echo time 39 ms; slice thickness 3 mm; number of slices 30; bandwidth 250 Hz/pixel; flip angle 150; matrix 448 × 448; field of view 16 cm), with in-phase image, and fat-suppressed water image; and (3) axial intermediate-weighted fat-suppressed sequence (repetition time 4990 ms; echo time 40 ms; slice thickness 2.5 mm; number of slices 39; bandwidth 150 Hz/pixel; flip angle 180; matrix 384 × 384; field of view 15 cm).

#### Measurement methods

The complete dataset was further analyzed based on the following models and measurement methods:

1. 3D surface model of medial and lateral meniscus;
2. 3D-calculated meniscal width, length, and height;
3. 2D-calculated meniscal width and length in MRI; and
4. 2D-derived meniscal width and length in RX.
5. 3D surface model of medial and lateral meniscus

3D surface models were created, as described in a previous paper [40], using the Materialise Interactive Medical Control System (MIMICS) 18.0 3D reconstruction software program (Materialise, Leuven, Belgium). The segmentation of the medial and lateral meniscus was manually performed by two trained orthopedic surgeons in sagittal and coronal slides (biplanar). Finally, all 3D surface models were smoothed (gap closing distance 0.0 mm, smallest detail 1.0 mm), and all left menisci were mirrored to the right surface models.

**Fig. 1:**
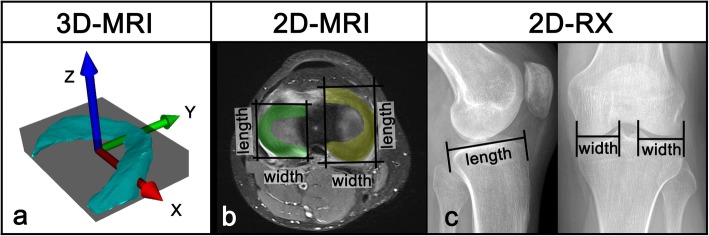
Measurement of meniscal width/length/height. **a** Width/length/height: meniscal dimensions are measured using an oriented bounding box. The box was aligned to the meniscus roots, and the size was adjusted as long as the entire meniscus body was enclosed. The dimensions of the meniscus correspond to the length of the sides of the box: *y*-axis (green arrow) = meniscal width; *x*-axis (red arrow) = meniscal length; *z*-axis (blue arrow) = meniscal height. **b** 2D-MRI sizing based on MRI of the contralateral side. **c** 2D-RX sizing according to Pollard on ipsilateral radiography

2) 3D-calculated meniscal width, length, and height (Fig. 1a)

The 3D measurement of meniscal width, length, and height was performed in the same way as described in a previous paper [40] with an oriented bounding box (= minimal-volume rectangular box that fully encloses the meniscal model) using the in-house developed planning software CASPA (Computed Assisted Surgery Planning Application, Balgrist CARD AG). The box was initially automatically generated around the 3D models. Then, the box was aligned parallel to a line from the anterior to the posterior meniscus root by rotation around the *z*-axis. Finally, the box dimensions were adjusted as long as the entire meniscus body was enclosed. Meniscus width (*y*-axis), length (*x*-axis), and height (*z*-axis) were now represented by the dimensions of the bounding box.

3) 2D-calculated width and length in MRI (Fig. 1b)

For the measurement of the 2D meniscal width and length in MRI, we used the following method. The measurements were performed on axial slides with reference to sagittal slides. Anterior and posterior meniscus roots were identified and connected by a first line. Parallel to this, a second line was placed adjacent to the outer contour of the meniscal base. Two additional lines, perpendicular to the first two lines, were set on the outer contours of the meniscus. Thereby, a 2D bounding box was created. The width and length of the meniscus corresponded to the dimensions of the box and could be measured.

4) 2D-derived width and length in RX (Fig. 1c)

The tibial plateau width was measured in the AP view perpendicular to the joint line as the distance between the margin of the tibial metaphysis to the medial lateral tibial eminence. According to Pollard, meniscal width is supposed to be equal to the tibia plateau width for medial and lateral menisci [31]. Tibia plateau length was measured in the LAT view perpendicular to the joint line as the distance between the anterior surface of the tibia above the tuberosity to the posterior margin of the tibia plateau. According to Pollard, the medial and lateral meniscal lengths can be calculated as 80% and 70%, respectively, of the tibia plateau length (medial meniscal length = 0.8 × tibia plateau length; lateral meniscal length = 0.7 × tibia plateau length).

### Part 2: Sizing methods

For the 3D-MRI meniscus-sizing method and for the 2D-MRI meniscus-sizing method, we used the contralateral meniscus as a template [40]. Therefore, both sizing methods are direct sizing methods, i.e., they were compared with the size of the meniscus on the contralateral side. For the 2D-RX sizing method, sizing was performed using an indirect calculation based on the bony anatomy of the same side [31].

**Fig. 2:**
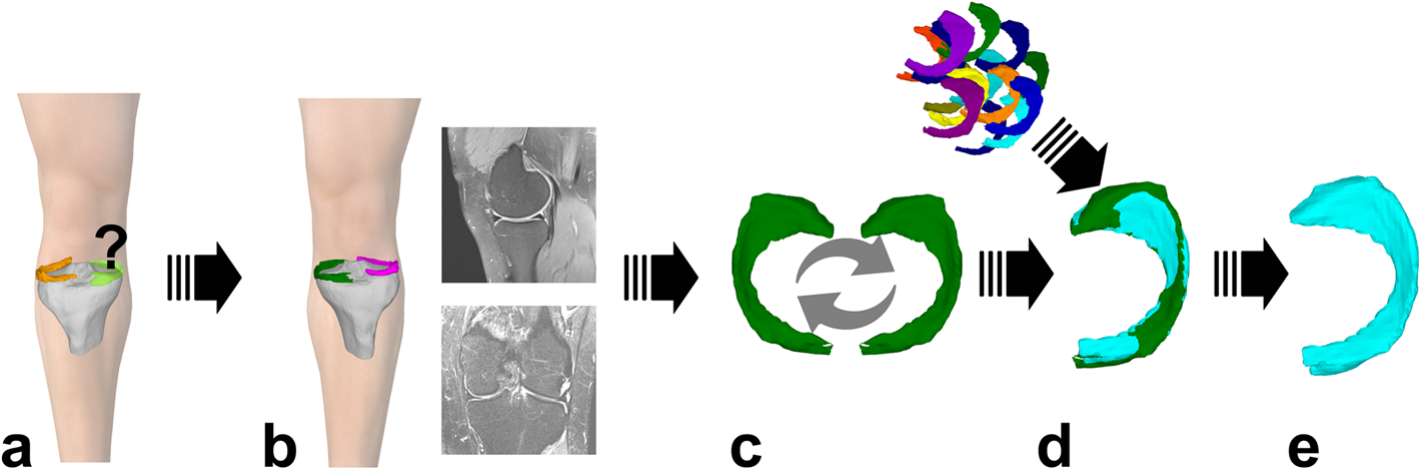
3D meniscus sizing (3D-MRI sizing). **a** Right knee with missing medial meniscus. **b** MRI scan of the contralateral side. **c** Meniscus segmentation and mirroring (see also Fig. 1). **d** Meniscus matching by all menisci in the tissue bank by mean and maximum surface distances (see also Fig. 3). **e** Selection of best fitting meniscus for meniscus allograft surgery

**Fig. 3:**
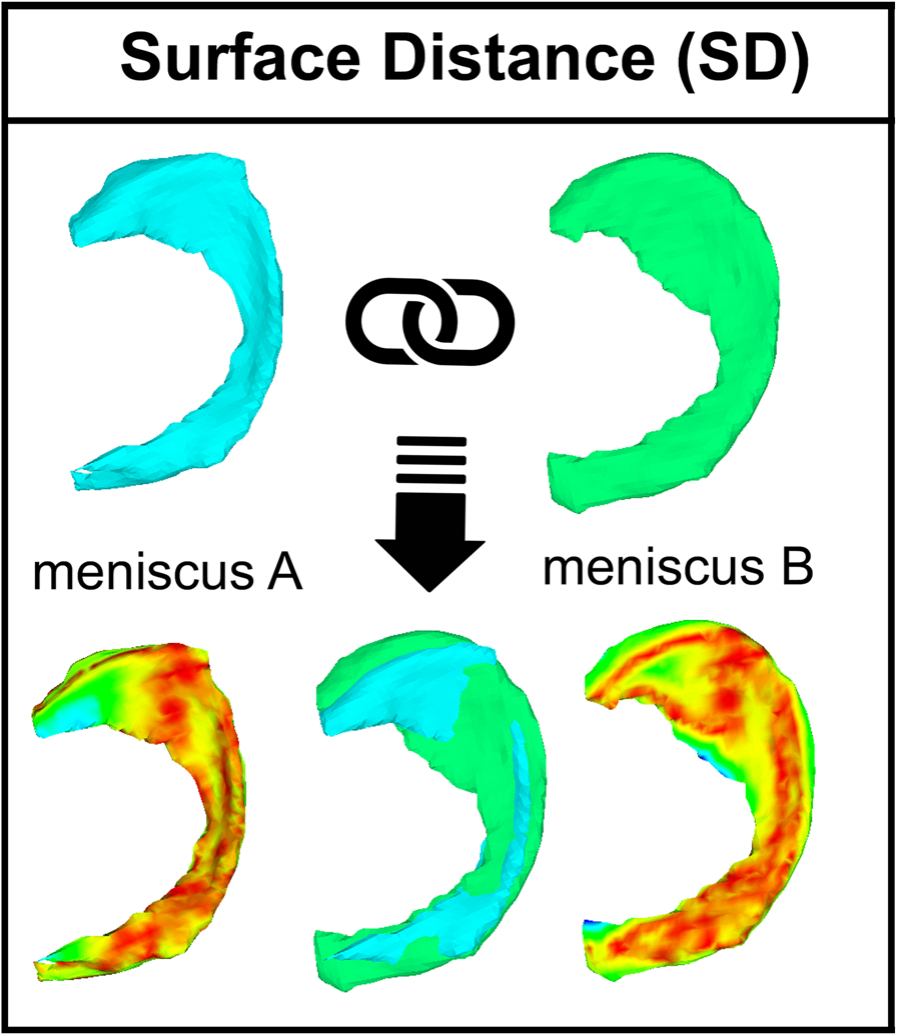
Surface distance (SD). The SD between two 3D models can be calculated after automatic superimposition by using the iterative closest point algorithm so that the distances of all surface points from one model to the other (and vice versa) are as small as possible. Maximum SD (MaSD) is calculated as the widest distance between “meniscus A” and “meniscus B.” The mean SD (MeSD) is the mean value of all surface points between “meniscus A” and “meniscus B.” For this analysis, the Hausdorff distance, i.e., the highest values, is used

1. 3D-MRI meniscus sizing (Fig. 2)

- Principle: Direct meniscus sizing, based on the mean surface distance (MeSD) of the contralateral meniscus.
- Requirements: MRI scan of the healthy contralateral knee and 3D surface models of the allografts.
- Surface distance: The similarity of two objects can be represented by the closest surface point distance as previously described [40]. To this end, the objects were automatically superimposed by using the iterative closest point (ICP) algorithm so that the distances of all surface points from one object to the other (and vice versa) were as small as possible. The maximum surface distance (MaSD) is the widest measured distance, and the MeSD is the mean of all surface distances. For this analysis, the Hausdorff distance, the highest distance value between the two objects, was used (Fig. 3).
- Allograft selection: The best fitting allografts were selected based on the closest MeSD values between the 3D template and all available 3D allograft surface models.

2) 2D-MRI meniscus sizing

- Principle: Direct meniscus sizing, based on calculated meniscal width and length of the contralateral meniscus.
- Requirements: MRI scan of the healthy contralateral knee, and the measured width and length of the allografts.
- Allograft selection: The best fitting allografts were selected by the smallest differences in width and length between the calculated sizes and the available allografts.

3) 2D-RX meniscus sizing

- Principle: Indirect meniscus sizing, based on derived meniscal width and length calculated from the measurement of the ipsilateral tibia plateau according to Pollard [31].
- Requirements: Conventional radiography of AP and mediolateral view of the ipsilateral side with calibrators.
- Allograft selection: The best fitting allografts were selected by the smallest differences in width and length between the calculated sizes and the available allografts.

### Part 3: Sizing validation

First, different terms must be clearly defined for better understanding. The missing meniscus—i.e., after meniscectomy—is hereby called the “original meniscus.” The 3D meniscus surface model for meniscus sizing is hereby called the “template meniscus.” The best fitting meniscus that was selected by a particular sizing method is hereby called the “selected meniscus.” In other words, we used different sizing methods of the template meniscus to choose the selected meniscus and then compared the selected meniscus with the original meniscus.

**Fig. 4:**
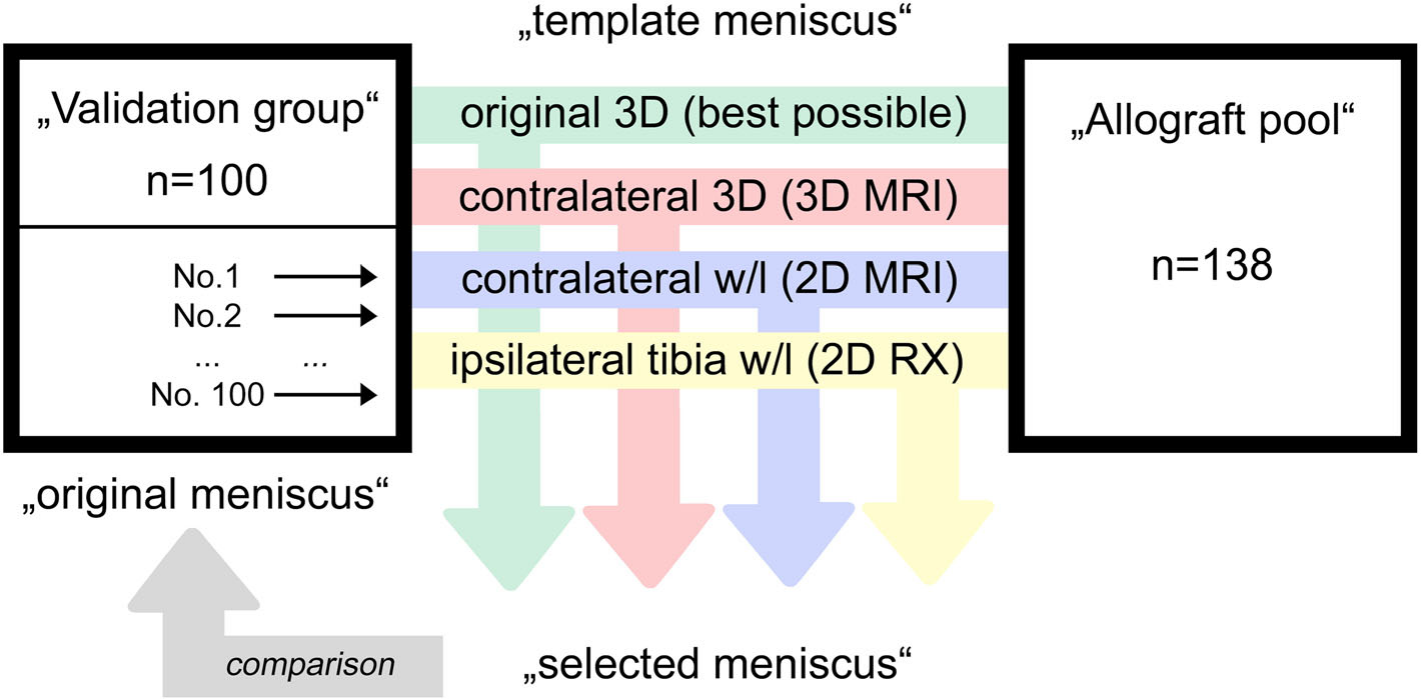
Validation. The validation was repeated for each of the 100 menisci from the 50 patients as follows (“validation group”): meniscus No. 1 of patient No. 1 was removed from the available pool. The sizing was performed by the “template meniscus.” The 3D surface model of the ipsilateral side (“original meniscus”) served as the best possible allograft (gold standard). The 3D surface model of the contralateral side corresponded to the 3D-MRI sizing. The meniscal width/length of the contralateral side in MRI corresponded to the 2D-MRI sizing. The derived meniscal width/length by the ipsilateral tibia plateau in RX corresponded to the 2D-RX sizing method. The best match (“selected meniscus”) out of the “allograft pool” was selected based on the different “template menisci.” Finally, the “selected meniscus” was compared with the “original meniscus.” This procedure was repeated for all 100 menisci of the “validation group”

Because sizing with surface distances does not allow a simple comparison of two values (i.e., width and length) of the original and selected meniscus, as was done in previous studies, sizing validation had to be performed by a more complex simulation (see Fig. 4). Therefore, the 50 patients with bilateral imaging served as the “validation group,” resulting in 100 (= 50 right and 50 left) different medial and lateral menisci. An imaginary tissue bank (“allograft pool”) was composed of all 100 menisci, together with the menisci from the unilateral imaging of another 40 patients, resulting in 140 different menisci. The menisci of each tested patient in the validation group were excluded from the potential menisci that could be selected for them, which finally resulted in an allograft pool of 138 different medial and lateral menisci. Therefore, for the validation group, we needed bilateral MRI scans to compare the previously described sizing methods. For the additional patients, this was not necessary, and we aimed to have as many allografts as possible in our tissue bank.

The simulation was then performed for each meniscus (meniscus No. 1 to meniscus No. 100) of the validation group. An in-house developed computer program automatically calculated the closest MeSD and MaSD between all 100 original (validation group) and 138 different allografts (allograft pool). Overall, there were 13,800 possibilities for allograft selection by MeSD (= 100 originals × 138 allografts), and the 100 closest menisci served as the best possible allografts, which served as the gold standard. In a second step, the same validation was now performed by the contralateral meniscus surface model as the template, which corresponds to the 3D-MRI sizing. Of the 13,800 possibilities, only the best fitting allografts by this sizing method were selected. In a third step, validation was performed using the closest difference in measured width and length of the contralateral side in MRI (2D-MRI sizing) and 3D measured width and length. Thereby, width and length were equally weighted and selected by the lowest error sum of squares: (width_sized_−width_allograft_)^2^ + (length_sized_−length_allograft_)^2^. In a fourth step, the same procedure was repeated based on the closest difference in derived width and length of the ipsilateral side (2D-RX sizing).

Finally, the original meniscus was compared with the selected menisci resulting from the different sizing methods using the surface distance as well as the meniscal width, length, and height as similarity measurements.

Meniscus outliers were defined as those with a difference between the original and selected meniscus over 5 mm in width, over 5 mm in length, or over 4 mm in height.

### Inter-rater/intra-rater reliability

The first 30 knee joints—in alphabetic order based on patient names—were used to calculate interclass correlation. 3D surface models (3D-MRI sizing), as well as width/length in MRI (2D-MRI sizing) and X-ray (2D-RX sizing), were calculated by two trained orthopedic surgeons, as described above. Intra-rater reliability was calculated by the repetition of these measurements by one of the surgeons.

### Statistics

The statistical analysis was performed with SPSS (IBM Corp. IBM SPSS Statistics for Windows, version 24.0. Armonk, NY: IBM Corp.) Descriptive analyses and independent-sample *t* tests were performed to investigate the meniscal diversity. *P* values below 0.05 were considered statistically significant. The correlations between width/length and MeSD/MaSD were analyzed with a linear regression analysis (Pearson correlation coefficient). Inter-rater and intra-rater reliability was calculated using interclass correlation coefficients (ICCs) (two-way mixed, absolute agreement). We used scatterplots and boxplots for visual presentation.

## Results

### Meniscus diversity (of the allograft tissue bank)

Overall, the medial meniscus (*n* = 140) was on average 31.6-mm (standard deviation (Std) ± 3.3 mm) wide, 46.8-mm (Std ± 3.7 mm) long, and 9.3-mm (Std ± 1.4 mm) high. The correlation between meniscal width and length was poor (*R*^2^ = 0.310).

**Fig. 5:**
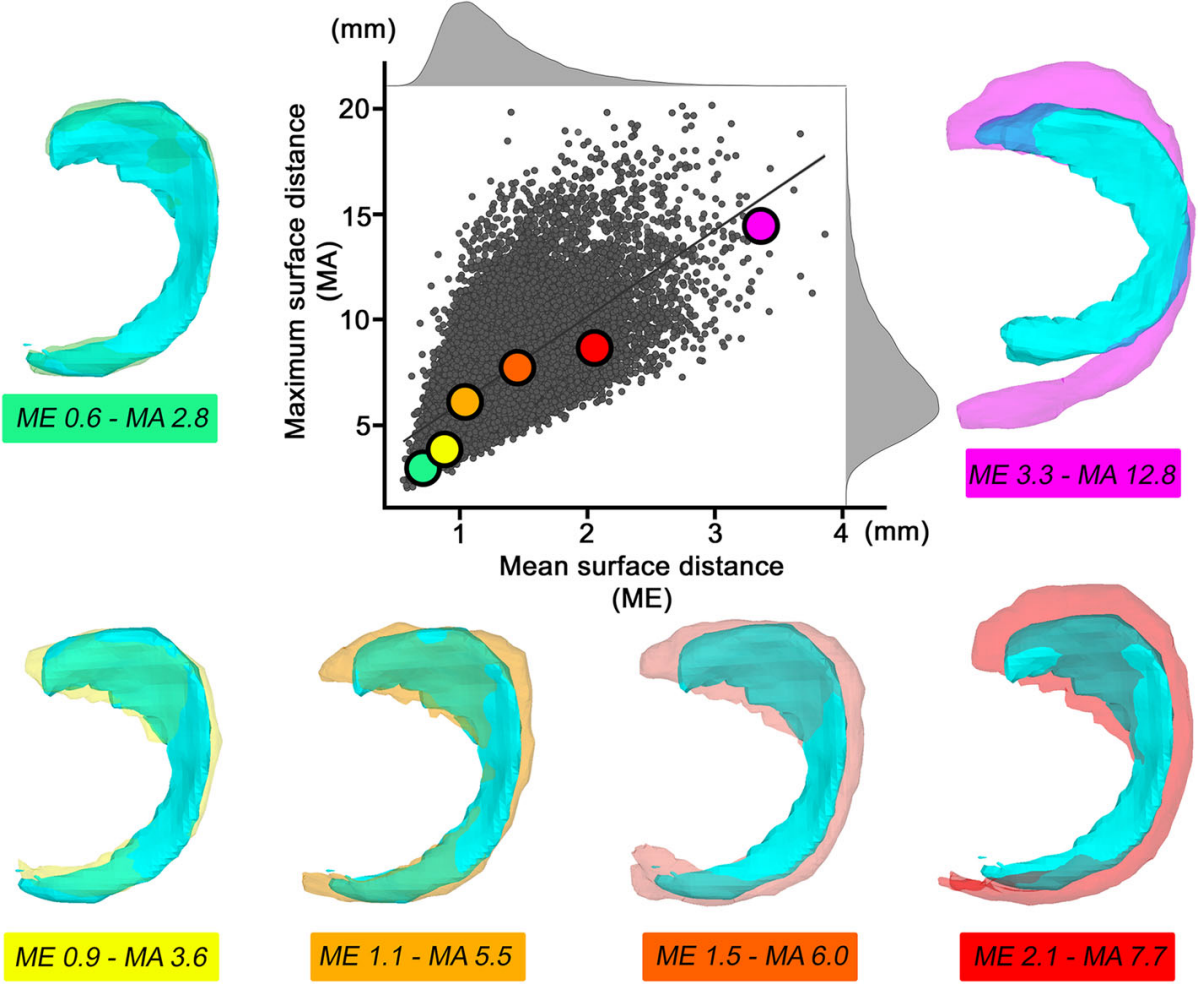
Correlations between the MeSD and MaSD values. Scatterplot of all 38,920 possibilities of medial and lateral meniscus combinations (38,920 = 140 × 139 for medial and 140 × 139 for lateral meniscus). MaSD = maximum surface distance; MeSD = mean surface distance. Six meniscus examples with increasing mismatch by increasing MaSD and MeSD values

The MeSD for all 140 medial surface models (*n* = 140 × 139 = 19,460) was on average 1.3 mm (Std ± 0.41 mm; min–max 0.57–3.70 mm), and the MaSD was on average 7.6 mm (Std ± 2.72 ; min–max 2.3–19.9 mm). The correlation between MeSD and MaSD values was *R*^2^ = 0.430 (Fig. 5).

The lateral meniscus (*n* = 140) was on average 31.7-mm (Std ± 3.7 mm) wide, 35.3-mm (Std ± 2.8 mm) long, and 9.9-mm (Std ± 1.4 mm) high. The correlation between meniscal width and length was poor (*R*^2^ = 0.304).

The MeSD for all lateral surface models (*n* = 140 × 139 = 19,460) was on average 1.4 mm (Std ± 0.45 mm; min–max 0.449–3.89 mm), and the MaSD was on average 7.1 mm (Std ± 2.55; min–max 2.00–20.2 mm). The correlation between MeSD and MaSD values was *R*^2^ = 0.495 (Fig. 5).

### Sizing validation

**Fig. 6:**
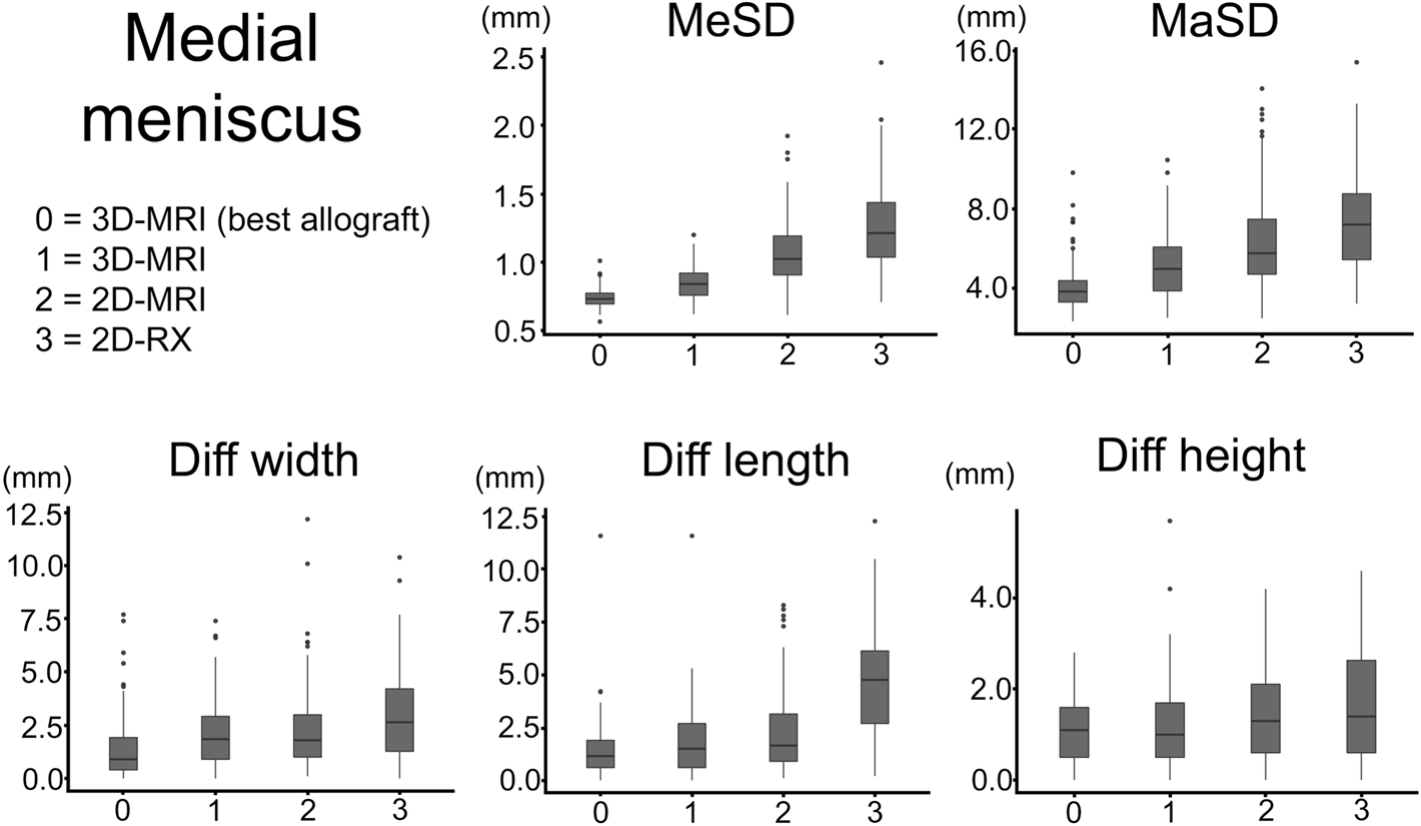
Results for the medial meniscus. Boxplots for the medial menisci. The median (middle quartile) marks the midpoint of the data and is shown by the line that divides the box into two parts. The box represents the middle 50% of values, and the upper and lower whiskers represent the range. The outliers are marked as separate points and defined as values more than 1.5 times the median. MeSD = mean surface distance; MaSD = maximum surface distance; Diff width/length/height= difference between the selected allograft dimensions and the original meniscus dimensions

**Fig. 7:**
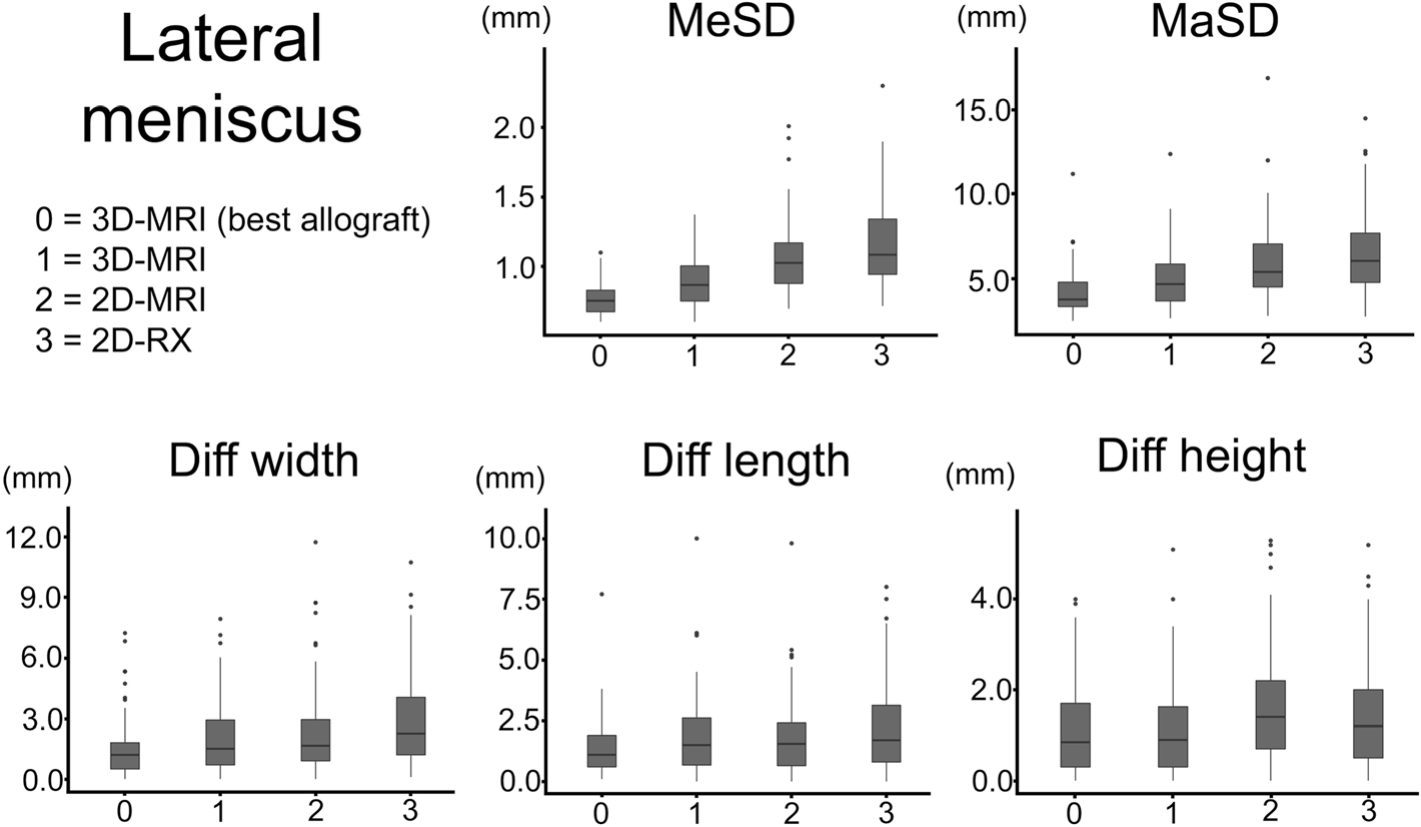
Results for the lateral meniscus. Boxplots for the lateral menisci. The median (middle quartile) marks the midpoint of the data and is shown by the line that divides the box into two parts. The box represents the middle 50% of values, and the upper and lower whiskers represent the range. The outliers are marked as separate points and defined as values more than 1.5 times the median. MeSD = mean surface distance; MaSD = maximum surface distance; Diff width/length/height= difference between the selected allograft dimensions and the original meniscus dimensions

All results are summarized in Tables 1 and 2 and Figs. 6 and 7.

**Table 1:** Results for the medial meniscus

Medial meniscus				
*N* =100	Best possible	3D MRI	2D MRI	2D RX
Mean surface distance (MeSD)				
Mean (mm)	0.74	0.85	1.07	1.27
Min–max (mm)	0.57–1.01	0.62–1.20	0.61–1.93	0.71–2.46
Std	0.07	0.12	0.25	0.34
*p* value best	–	0.001	< 0.001	< 0.001
*p* value 3D MRI	–	–	< 0.001	< 0.001
*p* value 2D MRI	–	–	–	< 0.001
Maximum surface distance (MaSD)				
Mean (mm)	4.09	5.12	6.47	7.52
Min–max (mm)	2.34–9.84	2.52–10.5	2.49–14.1	3.23–15.4
Std	1.26	1.61	2.43	2.53
*p* value best	–	0.002	< 0.001	< 0.001
*p* value 3D MRI	–	–	< 0.001	< 0.001
*p* value 2D MRI	–	–	–	0.002
Width difference				
Mean (mm)	1.5	2.1	2.4	2.9
Min–max (mm)	0–7.7	0–7.4	0.1–12.2	0–10.4
Std	1.6	1.5	2.0	2.2
*p* value best	–	0.126	0.006	< 0.001
*p* value 3D MRI	–	–	1.000	0.017
*p* value 2D MRI	–	–	–	0.275
Length difference				
Mean (mm)	1.4	1.8	2.3	4.7
Min–max (mm)	0–11.6	0–11.6	0.1–8.3	0.2–12.3
Std	1.4	1.7	2.0	2.6
*p* value best	–	0.911	0.009	< 0.001
*p* value 3D MRI	–	–	0.476	< 0.001
*p* value 2D MRI	–	–	–	< 0.001
Height difference				
Mean (mm)	1.1	1.2	1.5	1.7
Min–max (mm)	0–2.8	0–5.7	0–4.2	0–4.6
Std	0.7	1.0	1.1	1.3
*p* value best	–	1.000	0.056	< 0.001
*p* value 3D MRI	–	–	0.388	0.002
*p* value 2D MRI	–	–	–	0.531
Outliers*				
W/L/H	5	10	22	58
MaSD	0	5	17	58

*Std* standard deviation, *Min* minimum, *Max* maximum, *W* width, *L* length, *H* height, *MaSD* maximum surface distance

*Outliers were defined as *W* (width difference) > 5 mm, *L* (length difference) > 5 mm, *H* (height difference) > 4 mm, or MaSD (maximal surface distance) > 5 mm

**Table 2:** Results for the lateral meniscus

Lateral meniscus				
	Best possible	3D MRI	2D MRI	2D RX
Mean surface distance (MeSD)				
Mean (mm)	0.77	0.89	1.07	1.17
Min–max (mm)	0.611–1.10	0.611–1.38	0.701–2.01	0.720–2.29
Std	0.10	0.17	0.25	0.31
*p* value best	–	< 0.001	< 0.001	< 0.001
*p* value 3D MRI	–	–	< 0.001	< 0.001
*p* value 2D MRI	–	–	–	0.007
Maximum surface distance (MaSD)				
Mean (mm)	4.04	4.90	5.89	6.46
Min–max (mm)	2.43–11.2	2.59–12.3	2.73–16.8	2.69–14.5
Std	1.32	1.70	2.21	2.27
*p* value best	–	0.010	< 0.001	< 0.001
*p* value 3D MRI	–	–	0.002	< 0.001
*p* value 2D MRI	–	–	–	0.208
Width difference				
Mean (mm)	1.5	2.0	2.3	2.8
Min–max (mm)	0–7.2	0–7.9	0–11.7	0.1–10.7
Std	1.4	1.7	2.1	2.2
*p* value best	–	0.253	0.016	< 0.001
*p* value 3D MRI	–	–	1.000	0.015
*p* value 2D MRI	–	–	–	0.242
Length difference				
Mean (mm)	1.4	1.8	1.8	2.2
Min–max (mm)	0.1–7.7	0–10.0	0–9.8	0–8.0
Std	1.1	1.6	1.5	1.9
*p* value best	–	0.176	0.388	< 0.001
*p* value 3D MRI	–	–	1.000	0.475
*p* value 2D MRI	–	–	–	0.221
Height difference				
Mean (mm)	1.1	1.1	1.6	1.5
Min–max (mm)	0–4.0	0–5.1	0–5.3	0–5.2
Std	1.0	1.0	1.2	1.2
*p* value best	–	1.000	0.010	0.150
*p* value 3D MRI	–	–	0.009	0.133
*p* value 2D MRI	–	–	–	1.000
Outliers*				
W/L/H	6	11	18	25
MaSD	20	28	57	69

*Std* standard deviation, *Min* minimum, *Max* maximum, *W* width, *L* length, *H* height, *MaSD* maximum surface distance

*Outliers were defined as *W* (width difference) > 5 mm, *L* (length difference) > 5 mm, *H* (height difference) > 4 mm, or MaSD (maximal surface distance) > 5 mm

None of the presented sizing methods always selected the best available fitting allograft (which corresponded to the original meniscus) out of the imaginary tissue bank. In particular, the 3D shape (MeSD and MaSD) in these cases was different between the selected and the original meniscus, while the width, length, and height were not distinct.

3D-MRI sizing, which takes the 3D shape into account, significantly improved the accuracy of allograft selection compared with 2D-MRI (*p* < 0.001) and 2D-RX (*p* < 0.001) sizing.

Furthermore, 3D-MRI sizing reduced the number of outliers. For the medial meniscus, there were 12% fewer outliers selected compared with 2D-MRI sizing and 48% fewer outliers selected compared with 2D-RX sizing. For the lateral meniscus, there were 7% fewer outliers selected compared with 2D-MRI sizing and 14% fewer outliers selected compared with 2D-RX sizing.

### Inter-rater/intra-rater reliability

The interclass correlations for the medial and lateral menisci were good to excellent; see Table 3 for details (< 0.5 = poor, 0.5–0.75 = moderate, 0.75–0.9 = good, > 0.9 = excellent).

**Table 3:** Interclass correlations (ICCs)

	Width		Length		3D surface	
	Intra-rater	Inter-rater	Intra-rater	Inter-rater	Intra-rater	Inter-rater
Medial meniscus						
3D-MRI sizing	0.917	0.911	0.969	0.958	0.954	0.911
2D-MRI sizing	0.942	0.835	0.802	0.787		
2D-RX sizing	0.902	0.896	0.934	0.880		
Lateral meniscus						
3D-MRI sizing	0.901	0.922	0.957	0.967	0.890	0.896
2D-MRI sizing	0.782	0.703	0.954	0.897		
2D-RX sizing	0.893	0.847	0.934	0.880		

## Discussion

The most appropriate methods for meniscus sizing are still debated. Even under laboratory conditions, the most familiar radiographic method of Pollard had a standard error of prediction of ± 2.9 mm for meniscal width and ± 3.8 mm for meniscal length [31], which corresponds to a standard error of approximately 8%. Considering that the proposed 10% graft mismatch could cause relevant articular problems (i.e., 3- or 4-mm size difference based on meniscal sizes reported in the literature), the Pollard method seems to not be completely safe [17, 37]. Although MRI sizing seems to be superior to the radiological methods, 17% of the measured menisci in a cadaver study by Shaffer had differences of over 5 mm compared with the anatomical measurements [36]. Other authors have shown better results with an average error rate of 4.0–5.3% [35]. Because sizing is performed based on two variables (i.e., width and length), inaccuracy is probably magnified by the combination of both.

In a previous study, a new 3D meniscus sizing method was proposed, which was based on a healthy contralateral meniscus template to reduce inaccuracy [40]. For this sizing method, there are some basic prerequisites, which must be fulfilled and will be discussed in the following section. First, an easily determined, pose-invariant measurement parameter is required to accurately compare freely moveable 3D bodies. Second, the segmented 3D meniscus has to correspond with the actual 3D anatomical shape. Third, the contralateral meniscus has to be similar. Fourth, the benefits of a 3D sizing method must be compared with those of the currently used sizing methods. Fifth, the additional benefit has to be validated with improved clinical results.

### Basic prerequisites for 3D sizing

1. The similarity of 3D models can be evaluated by the surface distance, as already described in a previous study [40]. However, it is not yet known whether 3D sizing should be performed by the closest MeSD or by the closest MaSD, or a combination of these measures. Depending on the chosen criterion, the best allograft would be the selected according to the MeSD or to MaSD. Because there were poor correlations between these two values (*R*^2^ = 0.430–0.495), similar to the poor correlations between meniscal width and length (*R*^2^ = 304–310), these values could provide different information. The MeSD was generally less variable as the MaSD could be affected by one single surface point with a wide distance between the two models—for example, due to a partly segmented transverse or meniscotibial ligament. Therefore, we assessed sizing primarily based on the MeSD.
  The MaSD value was on average 5.3 times the MeSD value in our collection of 38,920 meniscus matched pairs. The best relationship was 2.6 times the value and reached up to 11.7 times the value. In conclusion, a very good fitting allograft could be expected by low (< 1.0 mm) MeSD combined with an MaSD value of less than 3 times the MeSD value. For examples, see Fig. 5.
2. A direct comparison of the anatomical and segmented shapes remains unavailable. However, to date, MRI measurements are often used as the gold standard and seem very good at identifying the meniscus tissue [32, 33, 35, 37, 39].
3. In a previous study, we showed that the contralateral side can be used as a very precise and reliable meniscus template for 3D sizing.
4. Our results are not directly comparable to previous studies. 3D sizing is based on MeSD and not on meniscal width and length. Therefore, a comparison to previous studies was not possible. We solved this problem by a simulated allograft selection based on the three sizing methods from an imaginary tissue bank of 138 different menisci. The selected allografts could be easily compared. An important advantage of this evaluation was the fact that the multiplicative inaccuracy due to a combination of two imprecise parameters (i.e., width and length) was taken into account. Herein, we showed a significant improvement of the 3D shape of the selected meniscus allograft with sizing by the MeSD compared with sizing by the combined meniscal width and length. There were also improvements based on meniscal width, length, and height measures, but these improvements were not statistically significant for all values. Similar to many other studies, the mean width and length values of the selected menisci were good and mostly acceptable across the three sizing methods. However, the main problem of meniscus allograft sizing is related to the number of selected outliers. In our opinion, this is exactly the strength of the 3D sizing method. The number of outliers selected could be reduced by 12–29% compared with 2D-MRI sizing and by 41–53% compared with 2D-RX sizing. We defined outliers as a size difference between the original and selected meniscus of > 5 mm in width or length and > 4 mm in height. A cutoff value of 5 mm for width and length has previously been used by other authors [25, 36]. A cutoff value based on meniscal height had not been previously described. In our collection, the meniscal height had a mean value of 9.3–9.9 mm with a standard deviation of only ± 1.4 mm. Therefore, a cutoff value for meniscal height of > 5 mm would probably never become applicable. Because the clinical evidence regarding meniscal height is still unclear, we did not choose our cutoff value based on the 10% meniscal mismatch rule [17], but on > 4 mm, which was slightly higher than the slide thickness and detected only 5 mismatched menisci overall.

There are few limitations of this study. As already mentioned under the second point above, a direct comparison of the anatomical and segmented shapes is missing. However, the meniscus can be accurately identified in MRI, and MRI is therefore often used as the gold standard [32, 33, 35, 37, 39]. Because a mismatch of 3–4 mm (10%) appears to be clinically relevant, a slide thickness of up to 3 mm can significantly influence the results. Therefore, a biplanar segmentation of the meniscus with excellent inter-rater and intrarater reliability for width and length (ICC_inter_ 0.913–0.973; ICC_intra_ 0.955–0.987) was used [40]. Furthermore, a larger tissue bank could probably have improved our results. However, the number of allografts used is probably sufficient, as there are many tissue banks with (much) less than 138 different available allografts (on request).

3D meniscus sizing has some great advantages, as already mentioned in a previous study [40]. In the present study, we demonstrated—even with a limited number of 138 allografts—that 3D sizing can significantly improve the accuracy and substantially reduce the number of relevant outliers. In conclusion, the limited number of meniscus allografts due to increased demand in the world could be much better matched to the patients. Moreover, this method can be used to do more than find the best fitting 3D allograft. If necessary, a slightly smaller size due to presumed extrusion or slightly greater size due to presumed shrinkage can be easily found by down- or upscaling the 3D template [14]. Furthermore, the feasibility of “bone plugs” or “bone bridge” fixation methods, which are supposed to be superior to “soft-tissue” and “suture bone tunnel” fixation techniques, could also be improved due to the 3D method identifying the shape of the meniscus [14, 42]. This 3D template could also be used in the future for biomimetic 3D-printed scaffolds [43, 44].

However, 3D sizing involves additional costs. An MRI scan of the contralateral side is needed, and the tissue banks must be willing to offer this option. 3D surface models must be generated for all allografts. In the future, meniscus segmentation could probably be performed in a semiautomated or fully automated manner [45, 46], which could reduce additional costs. Automatized comparison of the meniscus template of the healthy contralateral meniscus and all available allografts is already possible.

## Conclusion

3D-MRI sizing by MeSD using the contralateral meniscus as a reconstruction template can significantly improve the accuracy of meniscus allograft selection and reduce outliers compared with sizing that relies only on width and length in radiography and MRI. Using these methods, the limited number of available allografts could probably be more effectively distributed based on the rising demand in the world.

## Data Availability

The datasets during and/or analyzed during the current study available from the corresponding author on reasonable request.

## Abbreviations

2D/3D: Two-/three-dimensional
AP: Anteroposterior
IPC: Iterative closest point
LAT: Lateral
MaSD: Maximum surface distance
MeSD: Mean surface distance
MIMICS: Materialise Interactive Medical Control System
MRI: Magnetic resonance imaging
RX: Radiograph
Std: Standard deviation
